# Editorial: Metabolic Health in Normal and Abnormal Sleep

**DOI:** 10.3389/fendo.2020.00131

**Published:** 2020-03-11

**Authors:** David Kim, Camilla Miranda Hoyos, Babak Mokhlesi, Sushmita Pamidi, Jonathan C. Jun

**Affiliations:** ^1^Division of Pulmonary, Critical Care, Department of Medicine, Johns Hopkins University, Baltimore, MD, United States; ^2^CIRUS, Centre for Sleep and Chronobiology, Woolcock Institute of Medical Research, University of Sydney, Camperdown, NSW, Australia; ^3^Section of Pulmonary and Critical Care, Department of Medicine, University of Chicago, Chicago, IL, United States; ^4^Division of Respiratory Medicine, Department of Medicine, McGill University, Montreal, QC, Canada; ^5^School of Medicine, Johns Hopkins University, Baltimore, MD, United States

**Keywords:** metabolism, sleep, sleep apnea, obesity, circadian

Sleep comprises one third of the human lifespan and therefore has a significant impact on metabolism. There is a growing recognition that sleep disorders are associated with obesity, metabolic syndrome, and cardiovascular disease. This Research Topic, “Metabolic Health in Normal and Abnormal Sleep” contains a collection of studies that address complex interactions between sleep and metabolic health.

Obstructive sleep apnea (OSA) is a common disorder closely associated with cardiometabolic disease. Framnes and Arble write a timely review of the bidirectional relationship between OSA and metabolic disease (Framnes and Arble). First, they cover established impacts of obesity on the pathogenesis of OSA. Then, they review evidence for the reverse, whereby changes in metabolic/endocrine function can affect OSA in a weight-independent manner. In keeping with this theme, several studies in this collection examine associations between OSA, and glucose metabolism. Temple et al. examined whether associations between OSA and glucose metabolism are modified by sex. They recruited non-diabetic men and women from the community (*n* = 145) and performed polysomnography followed by a morning oral glucose tolerance test (OGTT) and derived measurements of insulin resistance and insulin response. In this cohort, 75% of men and 43% of the women had OSA. In those without OSA, they observed no sex differences in glucose metabolism. Among those with OSA, men exhibited higher glucose and insulin levels than women. The Authors ascribed their findings to OSA conferring a higher risk of diabetes in men (Temple et al.).

Fewer studies have examined the association of OSA and glucose metabolism in adolescents. Hannon et al. enrolled obese patients referred to a weight management clinic (age 12–18, BMI 38.9, *n* = 57) and performed polysomnography as well as OGTT. Thirty-six subjects had normal OGTT results while 21 exhibited impaired glucose tolerance or fasting hyperglycemia. Those with dysglycemia had higher body fat, but were not different in terms of OSA metrics. The Authors concluded that larger studies will be needed to clarify metabolic risks of OSA in obese youth.

One of the frequently cited mechanisms linking OSA to diabetes risk is exposure to intermittent hypoxia. Pham et al. examined this phenomenon by analyzing the pattern of oxygen desaturations in relation to frequently sampled overnight glucose levels in subjects during CPAP withdrawal (*n* = 30). They found that the depth of desaturation did not predict glucose elevation, whereas oxyhemoglobin “overshoots” mitigated hyperglycemia (Pham et al.). This suggests that the *pattern* of hypoxemia—not simply the average oxygen level—is a novel predictor of the glycemic response to OSA.

The liver is a target organ of metabolic syndrome, manifesting as non-alcoholic fatty liver disease (NAFLD). Scartabelli et al. examined associations between OSA and NAFLD, assessed using ultrasound in a cohort of obese women referred to an obesity center in Italy (*n* = 97, BMI = 50). Seventy patients had OSA, of which 53, 26, and 21% were classified as mild, moderate, and severe, respectively. Those with OSA were older, had greater liver size, intra-abdominal fat, and modestly elevated liver function tests levels compared to those without. Regression analysis showed expected associations between neck circumference, abdominal fat, and OSA severity. Liver size was also positively associated with AHI. These findings confirm liver enlargement as a marker of visceral adiposity but do not necessarily imply causation from OSA (Scartabelli et al.).

OSA is much more common in men than women, related to neuro-anatomical and hormonal differences. Morselli et al. examined whether this sex difference might also be related to slow-wave activity (SWA) during sleep, as (a) SWA is higher in women than age-matched men and (b) SWA transiently improves OSA, possibly due to favorable arousal threshold and airway collapsibility physiology. They performed polysomnography in 101 subjects (44 men, 57 women) and used spectral analysis to quantify SWA. As expected, OSA was much more common among the men with an adjusted OR = 3.17. Interestingly, AHI was inversely related to SWA in men, but not in women. Higher testosterone levels were independently associated with lower SWA after controlling for age, race, and AHI. The authors speculated that higher testosterone predisposes to lower SWA/arousal threshold, leading to greater ventilatory instability and greater risk for OSA in men (Morselli et al.).

OSA is also associated with gestational diabetes (GDM), although it is not clear whether OSA is causally implicated. Pamidi et al. plan to address this question by planning a randomized clinical trial to determine the feasibility and efficacy of CPAP (vs. nasal dilator placebo) to improve 24-hr glucose profiles in pregnant women (week 24–28 gestation) with OSA and GDM. The women will be followed until delivery and undergo OGTT at 12 weeks postpartum (Pamidi et al.).

Besides OSA, altered sleep duration and circadian rhythm are associated with obesity. This Research Topic also contains studies that examine this phenomenon. Anothisintawee enrolled 2133 Thai patients with prediabetes and used questionnaires to obtain Composite Scale of Morningness (CSM) score and self-reported sleep duration/timing. They found that evening preference (low CSM score) was associated with increased BMI and this relationship was mediated via reduced sleep duration. The novelty of their analysis is the implication that evening chronotype might be a modifiable risk factor for obesity, if insufficient sleep drives the association (Anothaisintawee et al.).

Titova et al. also examined the association between short sleep duration and adverse metabolic outcomes by examining the prevalence of metabolic syndrome in the Swedish EpiHealth cohort study (*n* = 19,691). They found increased prevalence of metabolic syndrome in those who reported either short sleep duration (< 6 h/day) or long sleep duration (≥9 h/day). The authors then stratified the analysis into middle-aged (45–65) and older (>65) subjects. Middle-aged subjects showed the same “U-shaped” curve of metabolic risk whereas in older subjects, only long sleep was linked to a higher prevalence of metabolic syndrome. This study reveals novel interactions between sleep duration and age on metabolic health (Titova et al.).

Gohil and Hannon contributed a related article reviewing the association between poor sleep and obesity in adolescents. They cite several cross-sectional studies showing that reduced sleep time, worse sleep quality, altered sleep architecture (reduced REM), delayed sleep phase, and social jetlag are each associated with increased obesity. They also discuss putative mechanisms including obesogenic food choices and changes in leptin and ghrelin signaling (Gohil and Hannon). Building upon the theme of sleep and its influence over appetite control, McHill et al. studied the impact of chronic insufficient sleep on appetite and related hormones. They enrolled 17 participants in a 32-days inpatient protocol that involved sleep restriction (4.6 vs. 6.67 h sleep) superimposed upon a 20-h day (forced desynchrony) to decouple timing of blood samples from the circadian phase. They observed a strong circadian pattern to hunger, leptin, ghrelin, insulin, and glucose, but did not find a differential impact of sleep restriction on this pattern. Although this was a “negative” study, it is positive in the sense that it highlights the powerful influence of circadian rhythm on metabolism (McHill et al.).

Pizinger et al. pose an important corollary question to several of the above studies: Does the literature support the concept that sleep *extension* improves cardiometabolic outcomes? They summarize studies that examined both the acute effects of sleep restriction and subsequent recovery on metabolic outcomes including leptin, glucose, insulin, and inflammatory profiles. In addition, they review a small number of interventional studies examining lifestyle changes, with or without a sleep extension component, and their impact on metabolic outcomes. Some studies found modest improvements in appetite, food intake, blood pressure, and insulin resistance. They conclude with a call to improve methodologies in this area, including a focus on short sleepers (Pizinger et al.).

Metabolism can also be viewed through the lens of energy expenditure, and sleep has a significant impact on metabolic rate. Chapman et al. provide a systematic review on the effect of insomnia on metabolic rate. Their literature search was confined to original studies of healthy adults with a comparator group, yielding only four articles (three from the same lab) evaluating 75 participants. In two studies, both day and night oxygen consumption was modestly elevated in those with insomnia compared to age/gender matched controls. One study showed no difference but only measured metabolic rate at one time point after awakening and included only females. The fourth study showed a minor reduction of metabolic rate during treatment of insomnia with lorazepam (Chapman et al.).

Lipid metabolism has been shown to affect sleep, and vice-versa. In particular, some studies show that fatty acids promote sleep, while sleep restriction increases fatty acids. Acyl-CoA synthetases are enzymes that bind intracellular fatty acids to CoA to form acyl-coA. The activated fatty acid is then able to undergo several metabolic or signaling fates. The Acyl-CoA synthetase pudgy (pdgy) is upregulated in starvation. Thimgan et al. evaluated the role of pdgy in Drosophila sleep. They determined that decreased expression of pdgy (transcriptional mutants) decreased baseline sleep, sleep rebound after sleep deprivation, survival during 48-h starvation in association with higher baseline heart rate and lower triglyceride levels. Similar results were observed with reduced pdgy transcription in fat bodies. The mechanism by which altered lipid metabolism curtails sleep is not known but could be related to impaired energy storage or utilization, signaling, or compensatory changes in endocrine axes (Thimgan et al.).

The articles in this e-book illustrate the importance of sleep toward healthy metabolic function, and the reciprocal effects of metabolic function on healthy sleep.

## Author Contributions

All authors listed have made a substantial, direct and intellectual contribution to the work, and approved it for publication.

### Conflict of Interest

The authors declare that the research was conducted in the absence of any commercial or financial relationships that could be construed as a potential conflict of interest.

